# Capecitabine-based chemotherapy in early-stage triple-negative breast cancer: a meta-analysis

**DOI:** 10.3389/fonc.2023.1245650

**Published:** 2023-10-25

**Authors:** Jie Bai, Xufeng Yao, Yinghong Pu, Xiaoyi Wang, Xinrong Luo

**Affiliations:** ^1^ Department of Breast and Thyroid Surgery, The First Affiliated Hospital of Chongqing Medical University, Chongqing, China; ^2^ Department of Head and Neck Surgery, The First Hospital of Jiaxing, Zhejiang, China

**Keywords:** capecitabine, triple-negative breast cancer, chemotherapy, survival, adverse events

## Abstract

**Introduction:**

The efficacy and safety of adjuvant capecitabine in early-stage triple-negative breast cancer remains undefined. A meta-analysis was conducted to elucidate whether capecitabine-based regimens could improve survival in early-stage triple-negative breast cancer (TNBC).

**Methods:**

The current study searched Medline, Embase, Cochrane Library, Web of Science, and ClinicalTrials.gov proceedings up to 2023.9. Disease-free survival (DFS), overall survival (OS), and grade 3–4 adverse events (AEs) were assessed. Extracted or calculated hazard ratios (HRs) and odds ratios (ORs) with 95% confidence intervals (CIs) were pooled.

**Results:**

The capecitabine-based regimens showed significant advantages in DFS (HR = 0.81, 95% CI: 0.73–0.90; *P* <.001) and OS (HR = 0.75, 95% CI: 0.65–0.87; *P* <.001) from 12 randomized controlled trials (RCTs) with 5,390 unselected participants. Subgroup analysis of DFS showed analogous results derived from patients with lymph node negative (HR = 0.68, 95% CI: 0.50–0.92; *P* = .006) and capecitabine duration no less than six cycles (HR = 0.73; 95% CI: 0.62-0.86; *P* <.001). Improvement of DFS in the addition group (HR = 0.77, 95% CI: 0.68–0.87; *P* <.001) and adjuvant setting (HR = 0.79, 95% CI: 0.70–0.89; P <.001) was observed. As to safety profile, capecitabine was associated with more frequent stomatitis (OR = 5.05, 95% CI: 1.45–17.65, *P* = .011), diarrhea (OR = 6.11, 95% CI: 2.12–17.56; P =.001), and hand–foot syndrome (OR = 31.82, 95% CI: 3.23–313.65, P = .003).

**Conclusions:**

Adjuvant capecitabine-based chemotherapy provided superior DFS and OS to early-stage TNBC. The benefits to DFS in selected patients with lymph node negative and the addition and extended duration of capecitabine were demonstrated.

## Introduction

Triple-negative breast cancer (TNBC) is characterized by aggressiveness, heterogeneity, and a higher relapse tendency. For histopathological diagnosis, it pertains to an estrogen receptor (ER), progesterone receptor (PR), and human epidermal growth factor receptor 2 (HER2) negative breast cancer (BC) subtype. Anthracycline- and/or taxane-based standard chemotherapies have substantially improved survival outcomes (1, 2). However, the 10-year recurrence risk in early-stage TNBC remains approximately 20%–40% (3, 4). Accordingly, new drug-incorporated strategies should be explored for further clinical development.

Capecitabine is an oral fluoropyrimidine prodrug with high efficacy and favorable tolerability for advanced BC (5). It is not the standard chemotherapy for early BC. There were also some conflicting survival data (6–17). Recently, two meta-analyses were performed to explore the RCTs of capecitabine effect, but TNBCs were treated as a subgroup in these studies (18, 19). To further determine the influence of adjuvant capecitabine on early-stage TNBC, we incorporated prospective randomized controlled trials (RCTs) in full text, performed a meta-analysis to get robust conclusions.

## Methods

### Search strategy

This meta-analysis complied with the Preferred Reporting Items for Systematic Reviews and Meta-Analyses (PRISMA) guidelines (20). Online databases Medline, Embase, Cochrane Library, and ClinicalTrials.gov were searched until September 17, 2023. Queries included the MeSH terms “breast cancer,” “breast neoplasm,” “triple-negative breast cancer,” “triple-negative breast neoplasms,” and the keywords “capecitabine” and “Xeloda.”.

### Selection criteria

Inclusion criteria were as follows: (a) phase III RCTs of early BC involving TNBC; (b) RCTs contained a comparison of capecitabine-based chemotherapy against capecitabine-free regimens; (c) available HRs with 95% CIs for DFS and/or OS. RCTs published other than English were excluded.

### Data extraction

Two reviewers (XL, JB) extracted data by search strategy independently. Discordance would be resolved by consensus. Information captured from RCTs included the following: trial name, authors, update year, study design, TNBC patients, baseline characteristics, chemotherapy schedules, median follow-up period, survival results (HRs and 95% Cis for DFS and/or OS), and grade 3–4 adverse events (AEs).

### Quality assessment

The quality of studies was independently assessed using the Cochrane Risk Of Bias Assessment Tool (CROBAT), which consists of “random sequence generation,” “allocation concealment,” “blinding of participants and personnel,” “blinding of outcome assessment,” “incomplete outcome data,” “selective reporting,” and “other bias” (**Figure S1**). Publication bias was evaluated by Funnel plot and Egger’s regression asymmetry test (21) (**Figures S2–S4**).

### Statistical analysis

By the generic inverse variance method, the HRs and 95% CIs for DFS and/or OS in TNBC were pooled (22).The odds ratios (ORs) of grade 3–4 AEs were weighted and estimated. In addition, current analysis followed the intention-to-treat principle. We conducted subgroup analyses in accordance with (a) nodal status, (b) capecitabine duration, (c) adding or replacing capecitabine in chemotherapy, (d) adjuvant or neoadjuvant chemotherapy, (e) dosage of capecitabine, (f) the TNBC proportion, (g) combined chemotherapy regimen, (h) sequential or concomitant capecitabine, (i) study region, (j) menopausal status, (k) tumor size, (l) histological grade, (m) basal or non-basal subtype, and (n) Ki-67 status. Heterogeneity among RCTs was evaluated by the Cochran Q statistics and I^2^ test (23). When *P* < 0.10 or I^2^ > 50%, we utilized the random-effect model. Otherwise, the fixed-effect model was used. Sensitivity analysis was conducted to assess the stability. Analyses were two-tailed with Stata 17.0 software.

## Results

### Characteristics of studies

**Figure 1:**
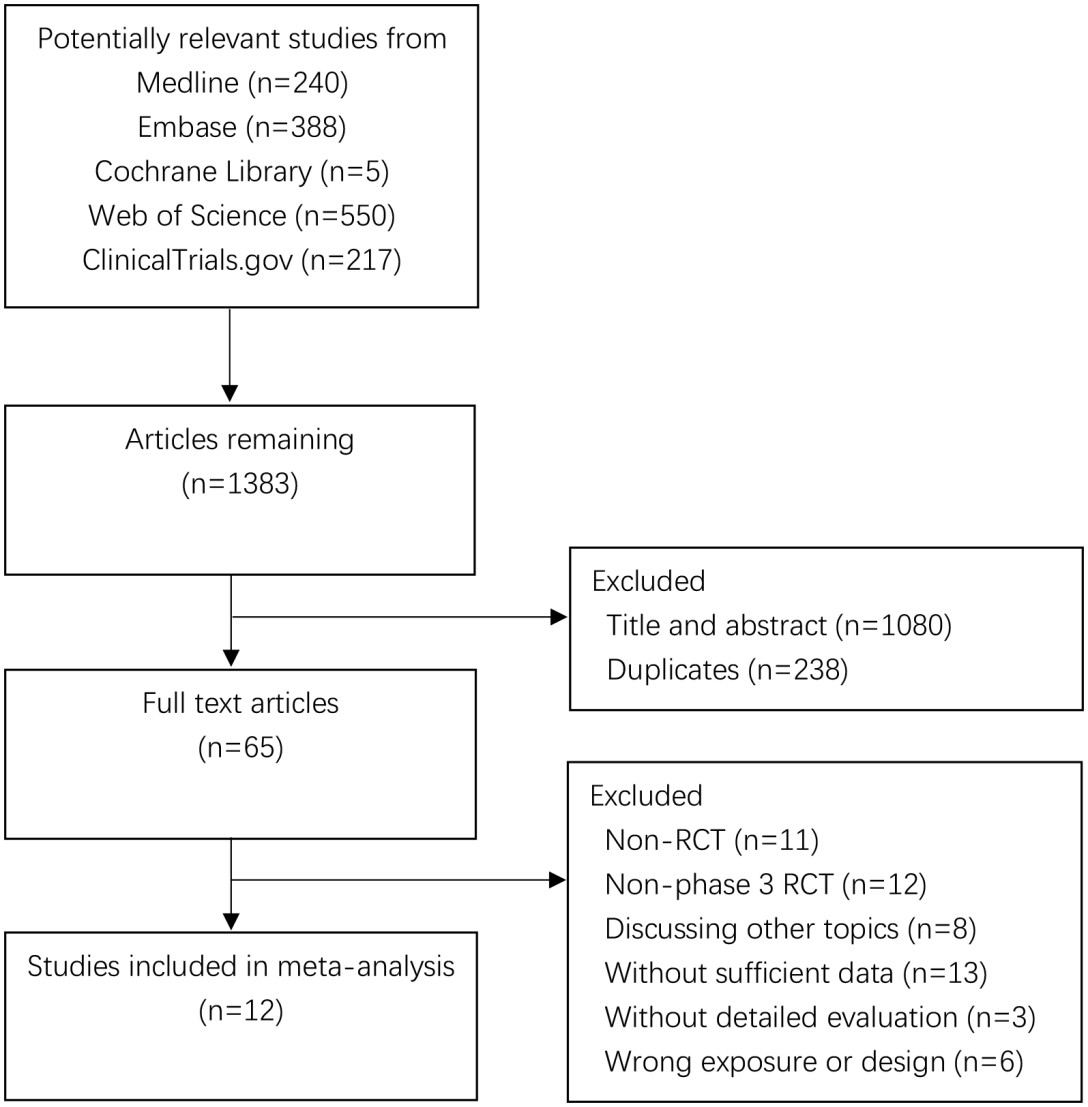
Flow diagram of the search process.

There were 12 RCTs with 5,390 TNBC patients who met the predefined criteria (**Table 1**; **Figure 1**) (6–17). The FinXX and CALGB 49907 trials, reported RFS rather than DFS; however, RFS was arguably the same definition as DFS (6, 11). Four studies reported early-stage TNBC only (7–10); nevertheless, the others incorporated all BC subtypes. The CBCSG010 trial conducted the modified intention-to-treat analysis, which might amplify the benefits, yet the dropouts were too small to yield a positive result (10).

**Table 1:** Study characteristics.

Study	Update year	Treatment		Age	TNM stage	TNBC, N (X/control)	Region	Median follow-up (years)	TNBC proportion	Design
		Capecitabine arm	Control arm							
FinXX	2022	3TX–3CEX	3T–3CEF	18-65	T_2-4_N_0_M_0_/ T_1-4_N_1-3_M_0_	93/109	Finland and Sweden	15	Subgroup	Adjuvant
EA1131	2021	NAC-6X	NAC-4Pla	≥18	T_2-4_N_0_M_0_/ T_1-4_N_1-3_M_0_	160/148	USA	1.7	Whole cohort	Neoadjuvant
SYSUCC-001	2021	Standard (neo) adjuvant chemotherapy-X (1 year, adjuvant)	Standard chemotherapy-observation	18-70	T_1c-3_N_0-2_M_0_	221/213	China	5.1	Whole cohort	Adjuvant
CIBOMA/2004-01	2020	Standard (neo) and/or adjuvant chemotherapy–8X	standard (neo) and/or adjuvant chemotherapy-observation	≥18	T_1-3_N_1-3_M_0_/ T_1c-3_N_0_M_0_	448/428	Spain and Latin America	7.3	Whole cohort	Neo/adjuvant
CBCSG010^†^	2020	3TX–3CEX	3T–3CEF	18-70	T_1-3_N_1-3_M_0_/ T_1c-3_N_0_M_0_	288/273	China	5.6	Whole cohort	Adjuvant
CALGB 49907	2019	6X	6CMF/4AC	≥65	T_1-3_N_1-3_M_0_/ T_1c-3_N_0_M_0_	76/78	USA	11.4	Subgroup	Adjuvant
CREATE–X	2017	Standard neoadjuvant chemotherapy– 6–8X (adjuvant)	Standard neoadjuvant chemotherapy-observation	20-74	T_1-4_N_1-2_M_0_/ T_1c-4_N_0_M_0_	139/147	Japan and Korea	5	Subgroup	Adjuvant
GAIN	2017	ddEC–PwX (4EC–10T4X)	iddEPC (3ETC)	18-65	T_1-3_N_1-3_M_0_	213/208	Germany	6.2	Subgroup	Adjuvant
TACT2	2017	4E–4X	4E–4CMF	≥18	T_0-3_N_0-2_M_0_	419/448	UK	7.1	Subgroup	Adjuvant
GEICAM/2003–10	2015	4ET–4X	4EC–4T	18-70	T_1-3_N_1-3_M_0_	95/71	Spain and Latin America	5	Subgroup	Adjuvant
USO 01062	2015	4AC–4TX	4AC–4T	18-70	T_1-3_N_1-2_M_0_/ T_1c-3_N_0_M_0_	396/384	USA	5	Subgroup	Adjuvant
GeparTrio	2013	2TAC-4NX	2TAC-4/6TAC	≥18	T_2-4_N_0_M_0_/ T_1-4_N_1-3_M_0_	362^‡^	Germany	5.2	Subgroup	Neoadjuvant

^†^The trial used the modified intention-to-treat (mITT) population, which included randomized patients received at least one dose of study drug.

^‡^The trial did not mention population of TNBC in the capecitabine arm or control arm.

X, capecitabine; C, cyclophosphamide; M, methotrexate; F, 5-fluorouracil; A, doxorubicin; T, docetaxel; E, epirubicin; P, paclitaxel; N, number; TNBC, triple-negative breast cancer; NAC, neoadjuvant chemotherapy.

There was no significant publication bias from the Funnel plot or Egger’s test (**Figures S2–S4**). Sensitivity analysis indicated that no certain trial affected the pooled results (**Figures S5, S6**).

### Efficacy

#### Pooled analysis

**Figure 2:**
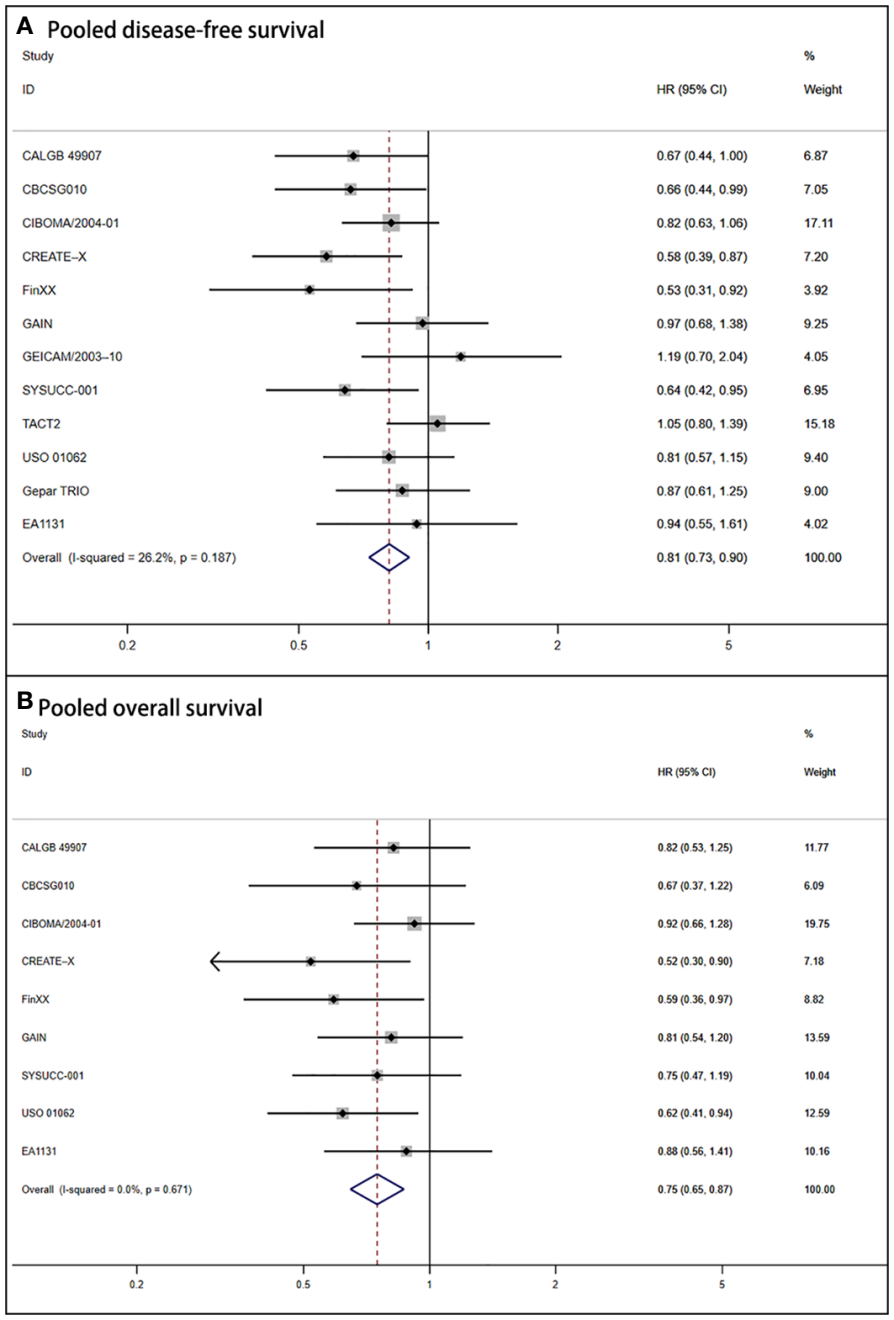
Forest plot of pooled disease-free survival and overall survival for capecitabine-based regimens in early triple-negative breast cancer **(A)** Shows the results for pooled disease-free survival. **(B)** Shows the outcomes for pooled overall survival.

Due to the absence of heterogeneity (*P* = .187; I^2 = ^26.2%), we utilized the fixed-effect model to calculate the pooled DFS (HR = 0.81, 95% CI: 0.73–0.90; P <.001). It also corresponded to significant improvement in OS for the capecitabine group (HR = 0.75, 95% CI: 0.65–0.87; P <.001). The forest plots of DFS and OS are shown in **Figure 2**.

#### Subgroup analysis

**Figure 3:**
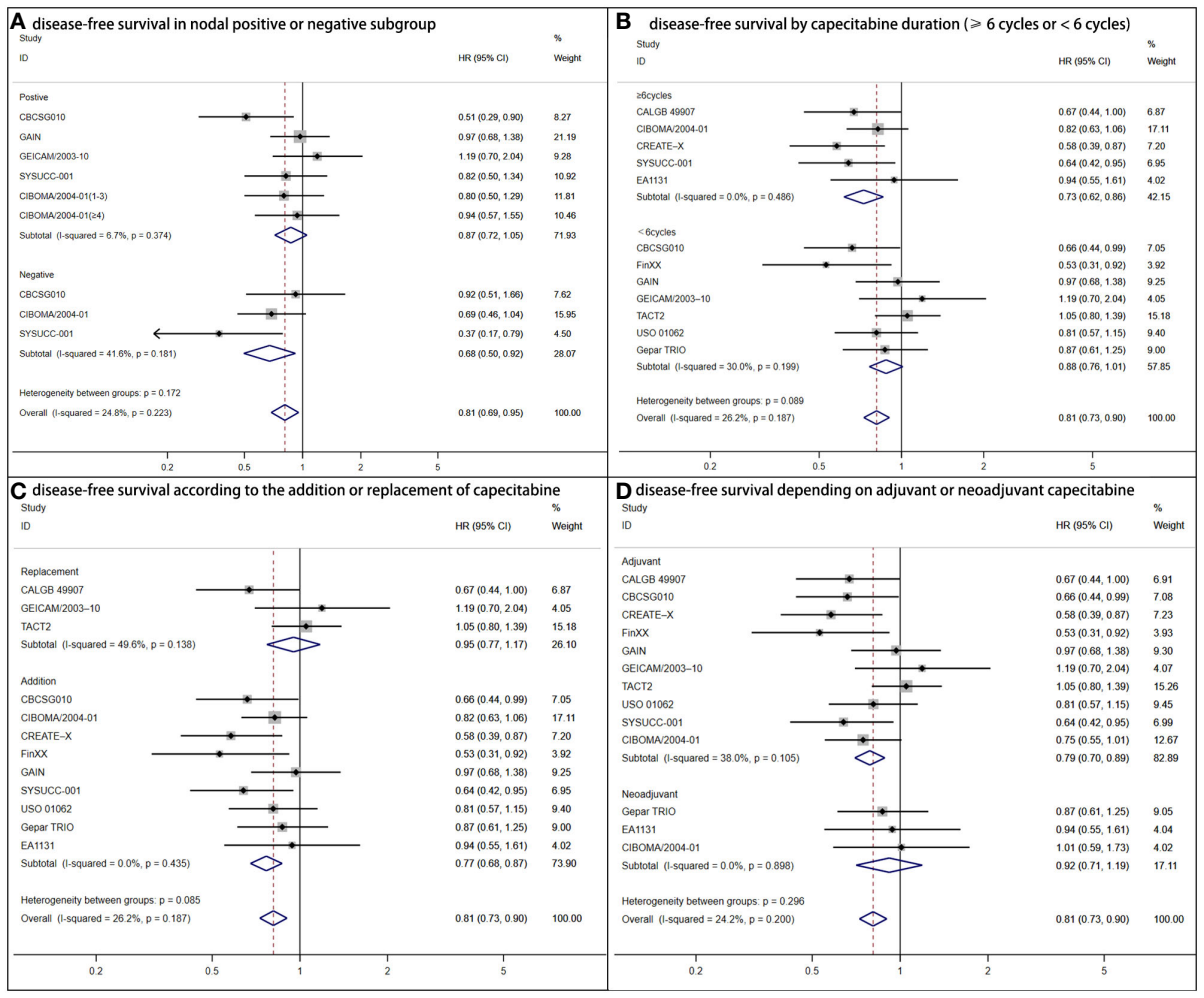
Forest plot of disease-free survival for adjuvant capecitabine in subgroups with heterogeneous results. **(A)** Shows disease-free survival in the nodal positive or negative subgroup. CIBOMA/2004-01(1-3) denotes subgroup with one to three positive lymph nodes. CIBOMA/2004-01(≥4) is defined as subset with no less than four positive nodes. **(B)** Shows disease-free survival by capecitabine duration (≥6 cycles or <6 cycles). **(C)** Shows disease-free survival according to the addition or replacement of capecitabine. **(D)** Shows disease-free survival depending on capecitabine accounting for adjuvant or neoadjuvant chemotherapy.

The nodal status subgroup analysis reached controversial outcomes (**Figure 3A**). DFS was statistically superior for women with lymph nodes negative (HR = 0.68, 95% CI: 0.50–0.92; *P* = .006) to positive (HR = 0.87, 95% CI: 0.72–1.05; *P* =.248). According to capecitabine duration, there were advantages of DFS (HR = 0.71, 95% CI: 0.60–0.84; *P* <.001) for patients who received capecitabine for at least 6 cycles (7, 9, 11, 12). For those with shorter duration (<6 cycles), no statistical significance was found (HR = 0.88, 95% CI: 0.73–1.01; *P* = .073) (**Figure 3B**) (6, 10, 13–17). The addition regimens improved DFS (HR = 0.77, 95% CI: 0.68–0.87; *P*<.001) (**Figure 3C**). In consideration of trials that TNBC acting as adjuvant chemotherapy, a greater DFS was determined (HR = 0.79, 95% CI: 0.70–0.89; *P* <.001) (**Figure 3D**) (6, 8–16).

As shown in **Table 2**, DFS was superior in the anthracycline and taxane arm (HR = 0.83, 95% CI: 0.71–0.97; P =.020) and non-basal subtype group (HR = 0.47, 95% CI: 0.31–0.71; P <.001). We observed no statistical significance in either menopause status. However, subgroup analysis for DFS yielded positive effects by capecitabine dosage (<1,000 mg or ≥1,000 mg), TNBC proportion (as subgroup or whole cohort), capecitabine sequence (in sequential or concurrent), study region (America–Europe or Asia), tumor size (≤2 cm or >2 cm), histological grade (1–2 or 3), and Ki-67 status (<30% or ≥30%).

**Table 2:** Subgroup analysis.

Subgroup	Variable	N^†^	HR	95%CI	P	I^2^ §	P for Q test §
Lymph node status							
Positive	DFS	5	0.87	0.72-1.05	0.152	6.7%	0.161
Negative			0.68	0.50-0.92	0.012	41.6%	0.355
Duration, cycle							
<6	DFS	12	0.88	0.73-1.01	0.073	30.0%	0.199
≥6			0.73	0.62-0.86	0.000	0.0%	0.486
<6	OS	9	0.68	0.53-0.85	0.001	0.0%	0.742
≥6			0.80	0.66-0.97	0.026	3.7%	0.510
Addition or replacement of capecitabine							
Addition	DFS	12	0.77	0.68-0.87	0.000	0.0%	0.435
Replacement			0.95	0.77-1.17	0.641	49.6%	0.138
Addition	OS	9	0.74	0.63-0.87	0.000	0.00%	0.588
Replacement			0.82	0.53-1.26	0.365	–	–
Chemotherapy							
Adjuvant	DFS	12	0.79	0.70-0.89	0.000	38.0%	0.105
Neoadjuvant			0.92	0.71-1.19	0.519	0.0%	0.898
Adjuvant	OS	8	0.71	0.59-0.86	0.000	0.0%	0.830
Neoadjuvant			0.69	0.41-1.16	0.159	51.6%	0.151
Dosage							
<1,000 mg	DFS	12	0.69	0.54-0.87	0.002	0.0%	0.399
≥1,000 mg			0.85	0.75-0.95	0.007	25.9%	0.214
<1,000 mg	OS	9	0.65	0.50-0.85	0.001	0.0%	0.755
≥1,000 mg			0.80	0.67-0.96	0.014	0.0%	0.613
TNBC proportion							
Subgroup	DFS	12	0.82	0.69-0.99	0.034	41.9%	0.099
Whole cohort			0.76	0.63-0.91	0.003	0.0%	0.557
Subgroup	OS	9	0.68	0.56-0.84	0.000	0.0%	0.577
Whole cohort			0.84	0.67-1.04	0.104	0.0%	0.775
Chemotherapy regimen							
Anthracycline without taxane	DFS	8	0.92	0.76-1.12	0.409	18.4%	0.298
Anthracycline and taxane			0.83	0.71-0.97	0.020	0.0%	0.470
Anthracycline without taxane	OS	3	0.62	0.41-0.94	0.024	–	–
Anthracycline and taxane			0.76	0.55-1.06	0.11	0.0%	0.604
Sequence							
Concurrent	DFS	12	0.77	0.63-0.94	0.010	26.5%	0.253
Sequential			0.83	0.73-0.94	0.004	33.2%	0.163
Concurrent	OS	9	0.68	0.54-0.85	0.001	0.0%	0.742
Sequential			0.80	0.66-0.97	0.026	0.0%	0.510
Menopause status							
Premenopausal	DFS	2	0.72	0.49-1.05	0.087	0.0%	0.790
Menopausal			0.72	0.36-1.18	0.190	54.8%	0.137
Region							
America–Europe	DFS	12	0.87	0.77-0.98	0.025	7.5%	0.373
Asia			0.63	0.50-0.79	0.000	0.0%	0.898
America–Europe	OS	9	0.78	0.66-0.92	0.005	0.0%	0.557
Asia			0.651	0.48-0.88	0.006	0.0%	0.604
Tumor size, cm^‡^							
≤2	DFS	2	0.52	0.31-0.88	0.014	0.0%	0.379
>2			0.68	0.47-0.84	0.035	0.0%	0.035
Histological grade							
1–2	DFS	2	0.52	0.29-0.94	0.032	0.0%	0.705
3			0.61	0.42-0.86	0.006	0.0%	0.437
Intrinsic subtype							
Basal subtype	DFS	3	0.93	0.76-1.12	0.434	0.0%	0.981
Non-basal subtype			0.47	0.31-0.71	0.000	45.5%	0.031
Ki-67							
<30%	DFS	2	0.53	0.28-0.98	0.044	0.0%	0.448
≥30%			0.71	0.51-0.98	0.039	0.0%	0.738

^†^The number of studies in the subgroup.

^‡^Tumor size was based on pathological assessment.

^§^Only one trial included, so the value of I^2^ and P for Q test are not available.

HR, hazard ratio; CI, confidence interval; DFS, disease-free survival; OS, overall survival.

#### Safety and tolerability

The safety profile included hematologic side effects, gastrointestinal events, general disorders, skin and subcutaneous disorders, nervous system disorders, investigations, musculoskeletal and connective disorders, vascular disorders, and other disorders (grades 3–4) (**Table 3**). It was shown in four-RCT-specified TNBC (7–10). The pooled results indicated higher frequencies of stomatitis (OR = 5.05, 95% CI: 1.45–17.65; P =.011), diarrhea (OR = 6.11, 95% CI: 2.12–17.56; P =.001), and hand–foot syndrome (OR = 31.82, 95% CI: 3.23–313.65; P =.003) for capecitabine in early TNBC women.

**Table 3:** Toxicity analysis.

Grade 3–4 AEs^†^	X arm (N = 1,114)	Control arm (N = 1,074)	OR	95% CI	p
Hematologic					
Leukopenia	1	1	0.98	0.06-15.63	0.986
Neutropenia	144	118	3.09	0.25-38.15	0.379
Thrombocytopenia	12	5	2.25	0.82-6.19	0.117
Gastrointestinal					
Nausea	10	5	1.85	0.66-5.23	0.421
Vomiting	13	15	0.84	0.41-1.74	0.182
stomatitis	15	3	5.05	1.45-17.65	0.011
Diarrhea	24	3	6.11	2.12-17.56	0.001
Abdominal pain	1	0	2.93	0.12-72.15	0.511
General disorders					
Fatigue	20	5	2.64	0.42-16.51	0.083
Hand–foot syndrome	124	3	31.82	3.23-313.65	0.003
Skin and subcutaneous disorders					
Rash	1	2	0.48	0.04-5.36	0.553
Alopecia	206	205	0.92	0.64-1.31	0.630
Nail changes	2	0	4.90	0.23-102.29	0.306
Nervous system disorders					
Sensory neuropathy	4	15	0.43	0.01-18.62	0.658
Investigations					
ALT and/or AST increase	8	10	1.33	0.19-9.32	0.123
Hyperbilirubinemia	3	0	6.87	0.35-133.42	0.203
Musculoskeletal and connective disorders					
Musculoskeletal pain (joint)	10	4	2.46	0.77-7.90	0.130
Musculoskeletal pain (muscle)	6	5	1.15	0.37-3.61	0.812
Vascular disorders					
Any cardiac event, general	2	1	1.95	0.18-21.63	0.585
Other					
Irregular menses	57	55	1.01	0.68-1.51	0.954

^†^Severity was based on the National Cancer Institute Common Terminology Criteria for Adverse Events (NCI-CTCAE) version 3.0 or 4.0.

AEs, adverse events; OR, odds ratio; CI, confidence interval; ALT, alanine aminotransferase; AST, aspartate transaminase.

## Discussion

There were several RCTs with conflicting results about capecitabine in early TNBC. Recently, the EA1131 and SYSUCC-001 trials provided updated outcomes with details about patient characteristics and treatment strategies (7, 8). In addition, the FinXX trial updated overall survival on the basis of approximately 15-year follow-up of the patients (6). Therefore, it is reasonable to reevaluate the influence of capecitabine. As for metastatic TNBC, capecitabine had low response rates and limited activity in trials (23, 24). Selecting appropriate patients may enhance treatment effects, since the mechanisms remained unclear.

Focusing on the association of capecitabine and early TNBC, there were two meta-analyses recently (18, 19). However, meta-analysis from Xun et al. did not extract data from the TACT2 trial, which should be included as well (14, 18). In addition, the GAIN and GEICAM/2003-10 trials only consisted node-positive patients; in other words, they should be incorporated to the nodal status subgroup analysis as well (13, 15). Zhou et al. included trials more rigorously, so subgroup analysis performed with less information (19).

We conducted a comprehensive meta-analysis of 12 RCTs (full text) exploring the efficacy and safety of capecitabine-based chemotherapy. It significantly improved DFS and OS among 5,390 TNBC patients. Considering subgroup analysis, the survival benefits were observed in lymph node negative status, the addition, extended duration, and adjuvant setting of capecitabine.

Patients with nodal negative results had a superior DFS. Regarding the node-positive arm, the CBCSG010 trial reported an advantage for DFS (10), whereas four RCTs (SYSUCC-001, CIBOMA/2004-01, GAIN, GEICAM/2003-10) did not (8, 9, 13, 15). In addition, there were three arms of nodal status (0, 1–3, and ≥4) in the CIBOMA/2004-01 trial (9). However, no more details were available for lymph node positive status (1–3, 4–9, ≥10, without metastasis to internal mammary chain or infraclavicular/supraclavicular region). Therefore, we could not further determine the influence of different positive-node stages.

Metastasis of lymph node contributes to higher risk for TNBC. We speculated that capecitabine would confer further extended survival on node-positive patients. Nevertheless, the result was paradoxical. It might be strong distinction of capecitabine on micro-metastatic and overt lesions. Targeting the immune escape metastasis mechanism, dormant tumor cells with a lower proliferation index were more sensitive to an antimetabolite drug. Similar to capecitabine, it is a DNA synthesis inhibitor (25). Biomarkers determining which TNBC subtype favored most are also needed; for example, the PAM50 non-basal molecular subtype and the BRCA1-like DNA copy number (26, 27). Extrapolation from studies of the last decade with long-term follow-up should be cautiously applied. The RCTs of individual survival benefits of capecitabine for node-positive patients are still needed.

Adjuvant capecitabine added to the anthracycline and taxane regimens had survival advantages for early TNBC, in favor of the synergism of docetaxel and capecitabine in preclinical models (28). The results verified the rationale of combination chemotherapy (29). Patients derived greater DFS from extended capecitabine duration (≥6 cycles), which means capecitabine could modulate antitumor immune and anti-angiogenesis properties through metronomic therapy (30).

Our study has limitations. First, populations with various characteristics contributed to the heterogeneity. The definition of ER-negative in the CBCSG010 and CREAT-X studies (<10%) did not align with others (<1%) (10, 12). Second, the diverse chemotherapy regimens confounded the results and decreased robustness. Third, given no individual patient data available, the details (age and intrinsic subtype) were incomplete, so it was hard to assess and select the subpopulation.

## Conclusion

This meta-analysis showed that capecitabine-based regimens significantly improved both DFS and OS in early-stage TNBC. There was a substantial improvement for DFS in the groups with lymph node negative status, the adjuvant, addition, and longer duration (≥6 cycles) of capecitabine to standard chemotherapy.

## Data availability statement

The original contributions presented in the study are included in the article/**Supplementary Material**. Further inquiries can be directed to the corresponding authors.

## Author contributions

Design: JB and XL. Data collection: JB, XY, YP. Data analysis: JB, XL. Writing-original draft: JB. Writing-review and editing: XW, XL. All authors contributed to the article and approved the submitted version.
