# Supplementary material for: Capecitabine-based chemotherapy in early-stage triple-negative breast cancer: a meta-analysis
Source: Front Oncol. 2023 Oct 25;13:1245650. doi: 10.3389/fonc.2023.1245650 (PMC10634425; doi:10.3389/fonc.2023.1245650)
Supplement: Supplementary file 1 [file Image_1.pdf]

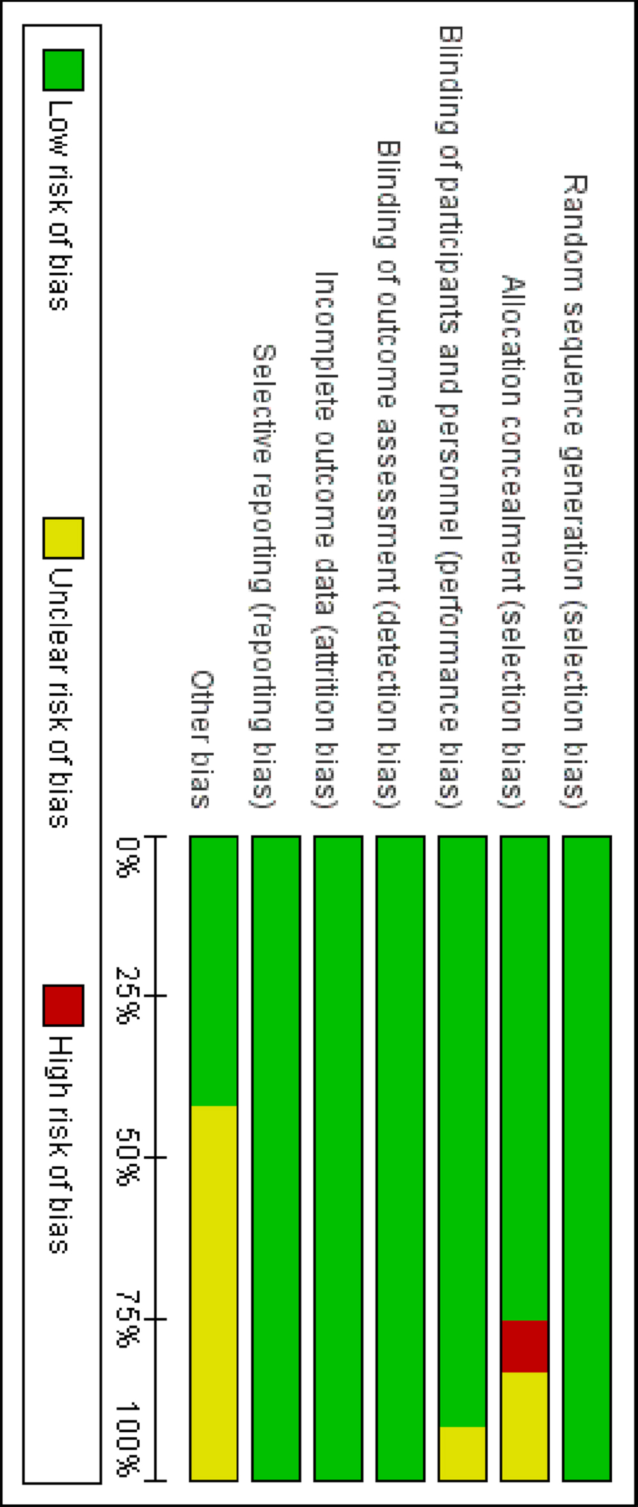
Low risk of bias
Unclear risk of bias
High risk of bias
Random sequence generation (selection bias)
Allocation concealment (selection bias)
Blinding of participants and personnel (performance bias)
Blinding of outcome assessment (detection bias)
Incomplete outcome data (attrition bias)
Selective reporting (reporting bias)
Other bias
0%
25%
50%
75%
100%

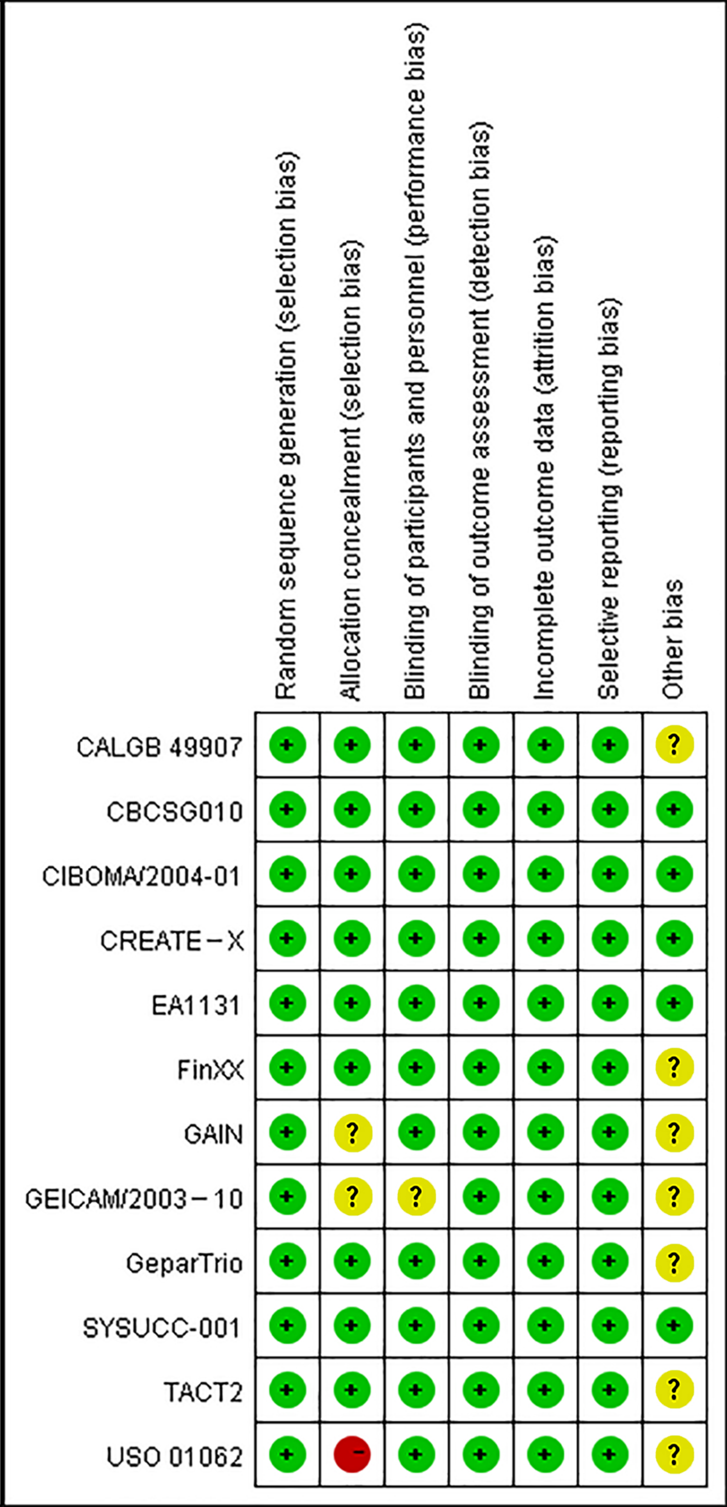
Random sequence generation (selection bias)
Allocation concealment (selection bias)
Blinding of participants and personnel (performance bias)
Blinding of outcome assessment (detection bias)
Incomplete outcome data (attrition bias)
Selective reporting (reporting bias)
Other bias
CALGB 49907
CBCSG010
CIBOMA/2004-01
CREATE−X
EA1131
FinXX
GAIN
GEICAM/2003−10
GeparTrio
SYSUCC-001
TACT2
USO 01062
