# Supplementary figures and images for: Capecitabine-based chemotherapy in early-stage triple-negative breast cancer: a meta-analysis
Source: Front Oncol. 2023 Oct 25;13:1245650. doi: 10.3389/fonc.2023.1245650 (PMC10634425; doi:10.3389/fonc.2023.1245650)

Funnel plot with pseudo 95% confidence limits

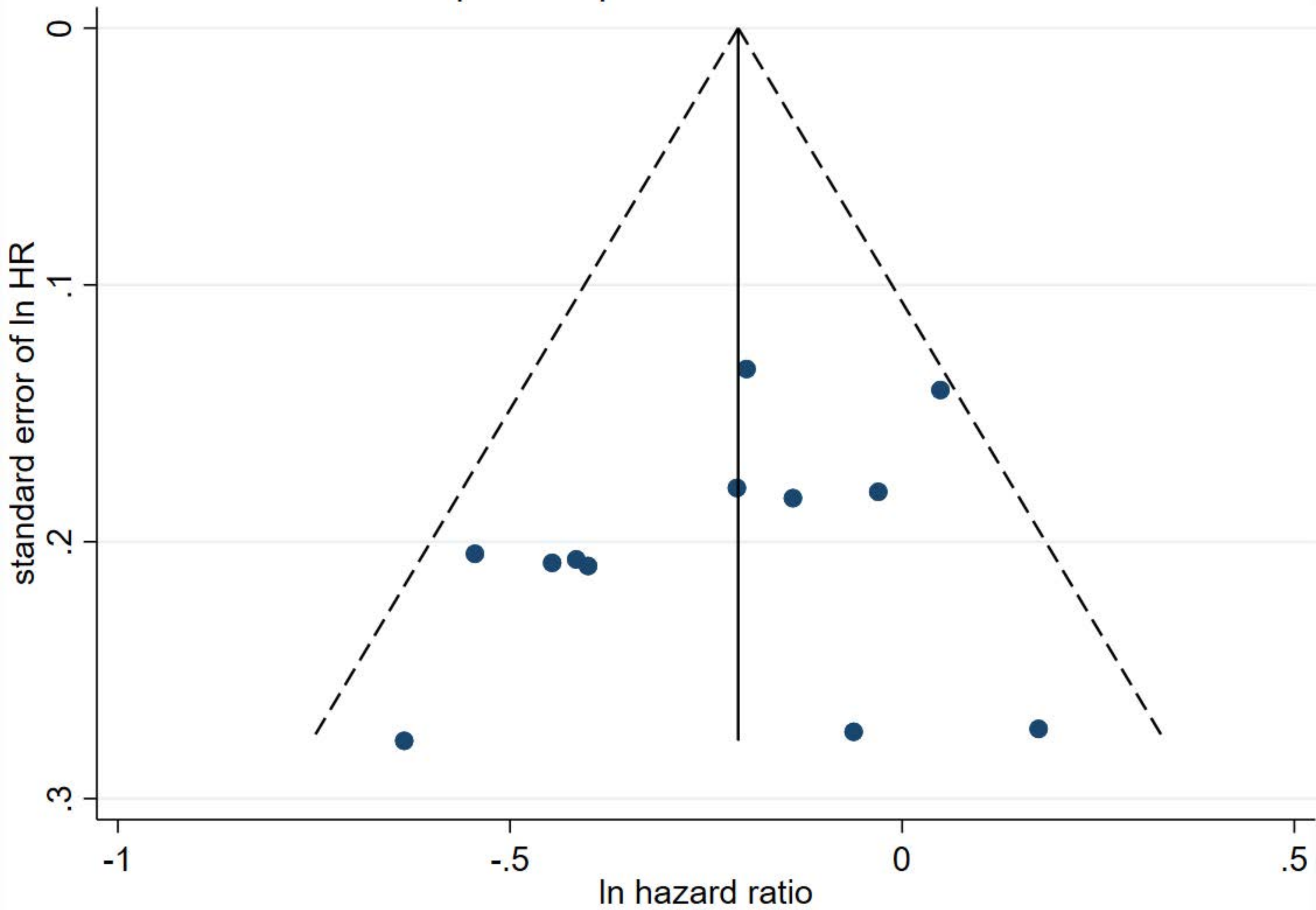

Supplement: Supplementary file 2 [file Image_2.pdf]

Funnel plot with pseudo 95% confidence limits

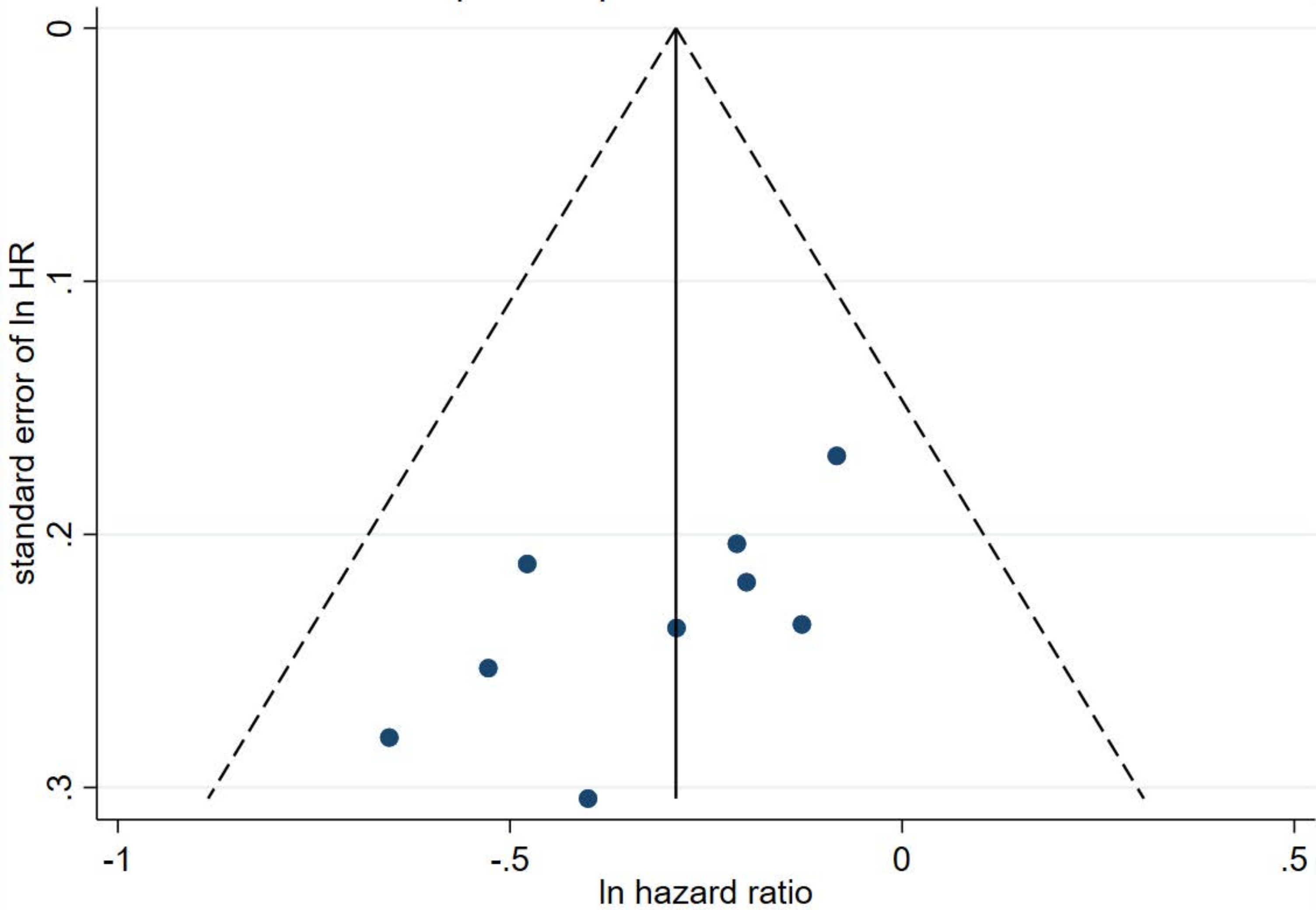

Supplement: Supplementary file 3 [file Image_3.pdf]

Egger's publication bias plot

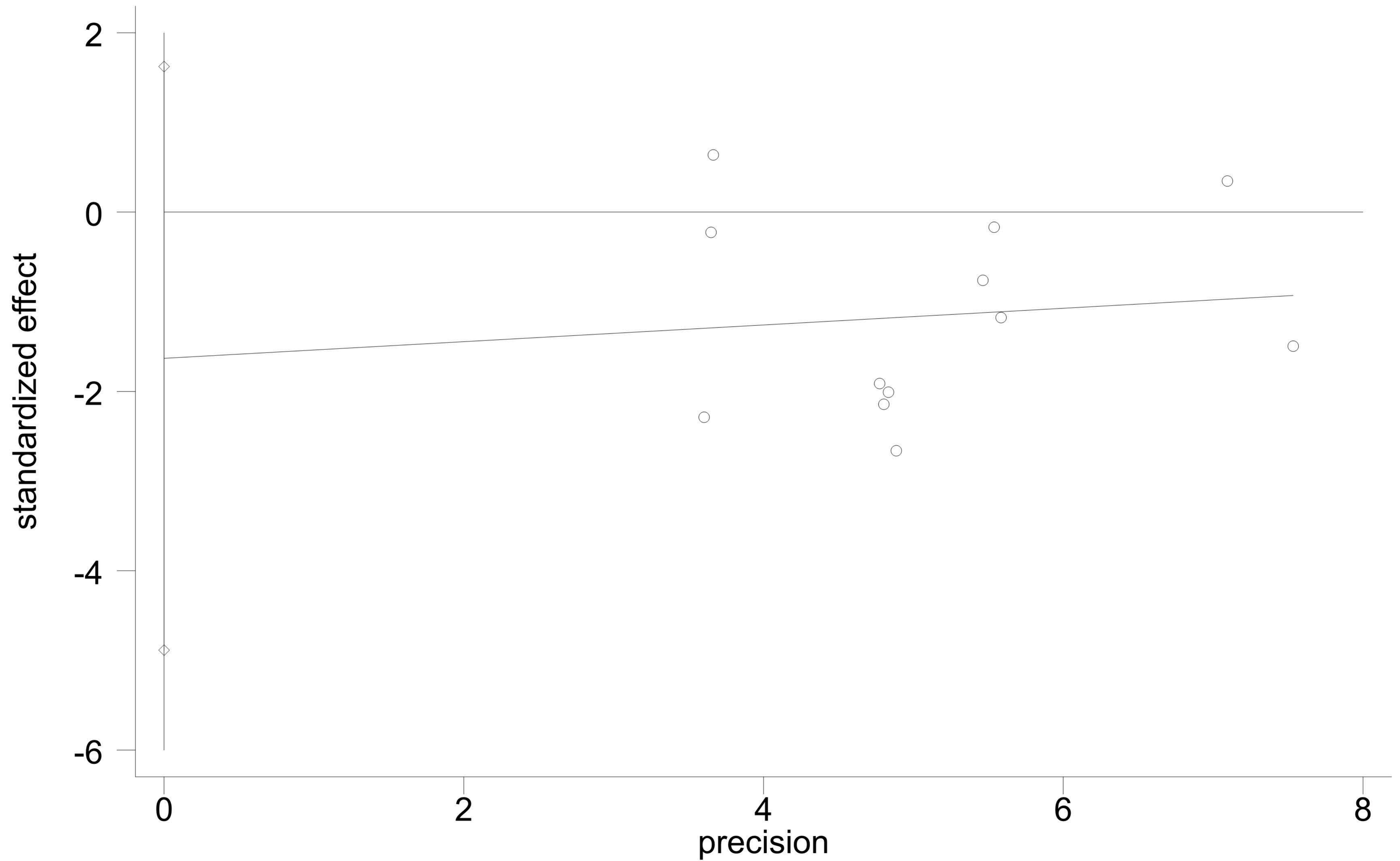

Supplement: Supplementary file 4 [file Image_4.pdf]

Meta-analysis estimates, given named study is omitted

| Lower CI Limit      ○ Estimate      | Upper CI Limit

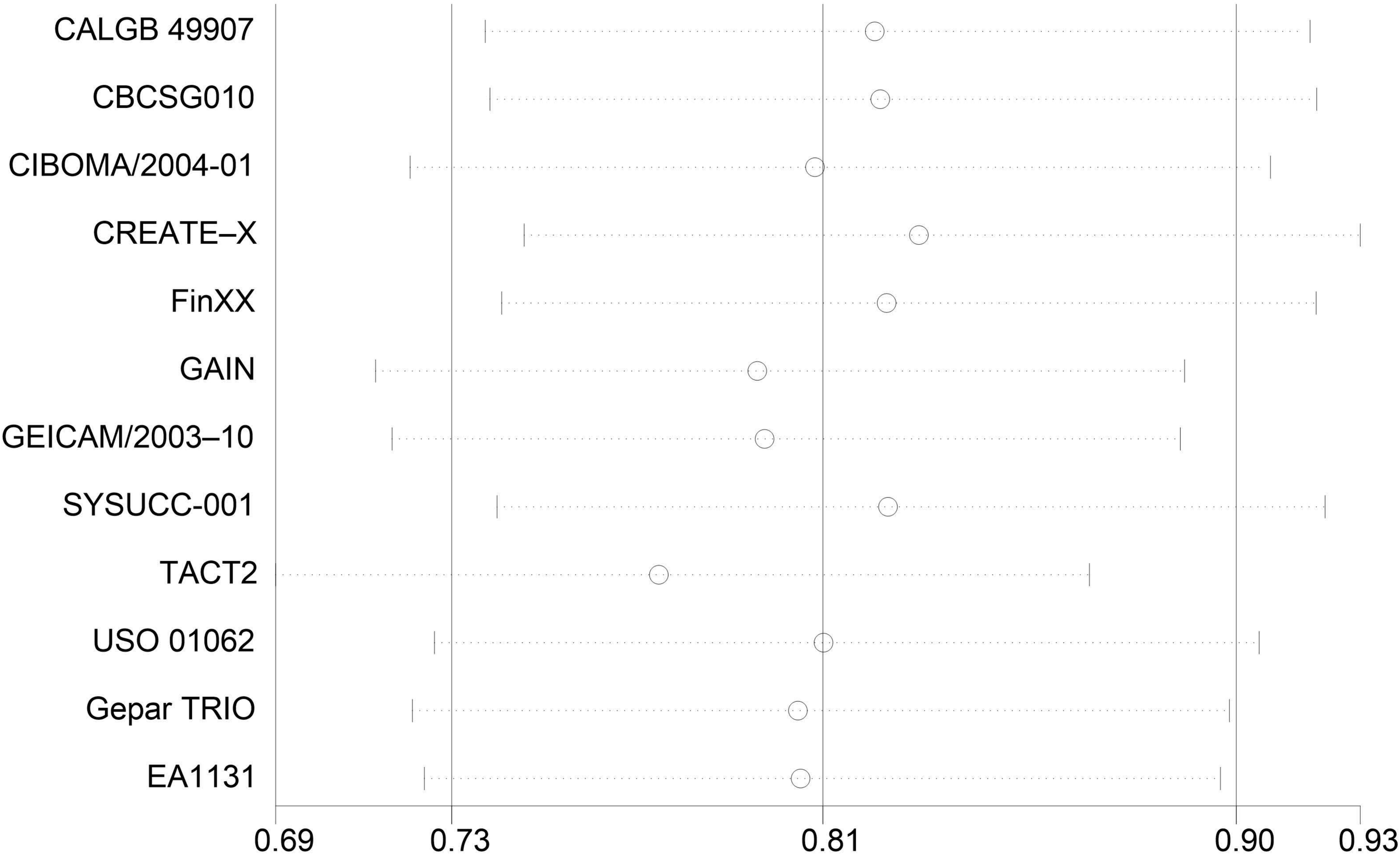

Supplement: Supplementary file 5 [file Image_5.pdf]

# Meta-analysis estimates, given named study is omitted

| Lower CI Limit

○ Estimate

| Upper CI Limit

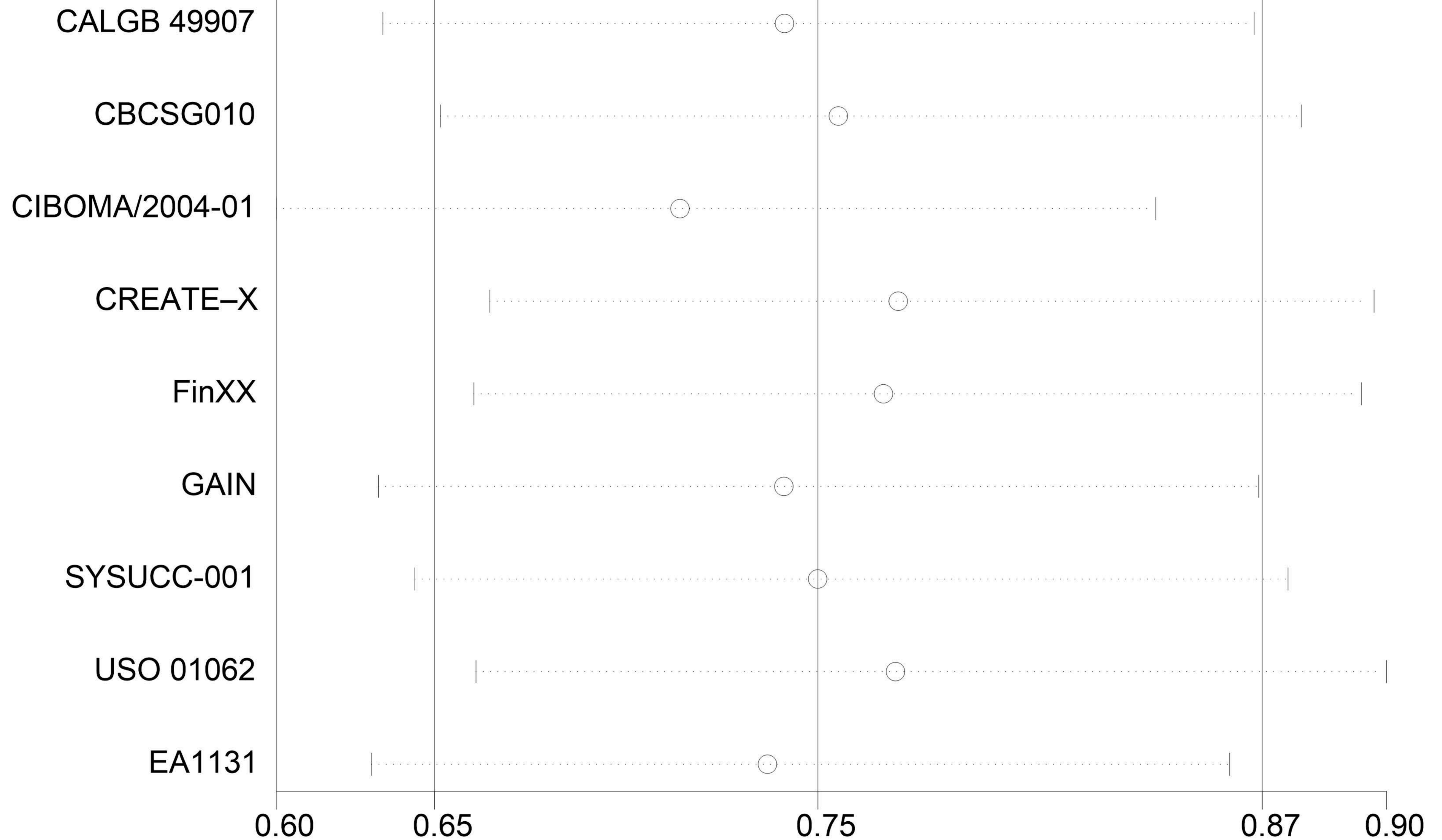

Supplement: Supplementary file 6 [file Image_6.pdf]
