# Supplementary material for: Capecitabine-based chemotherapy in early-stage triple-negative breast cancer: a meta-analysis
Source: Front Oncol. 2023 Oct 25;13:1245650. doi: 10.3389/fonc.2023.1245650 (PMC10634425; doi:10.3389/fonc.2023.1245650)
Supplement: Supplementary file 7 [file DataSheet_1.docx]

**Appendix**

The additional analyses specified in the statistical analysis plan are provided in the appendix to this supplement.

**Supplementary Figures**

**Figure S1.** Risk of bias graph and summary for included studies

Panel A shows risk of bias graph for included studies. Panel B shows risk of bias Risk for included studies.

**Figure S2.** Funnel of pooled disease-free survival for adjuvant capecitabine regimens in early-stage triple negative breast cancer

**Figure S3.** Funnel of pooled overall survival for adjuvant capecitabine regimens in early-stage triple negative breast cancer

**Figure S4.** Egger’s test for pooled disease-free survival of adjuvant capecitabine chemotherapy.

**Figure S5.** Sensitivity analysis for pooled disease-free survival with random-effect model

**Figure S6.** Sensitivity analysis for pooled overall survival with random-effect model

**Supplementary Table**

**Table S1.** Egger's test for pooled disease-free survival

| **Item** | **Coef.** | **SE** | **95%** **CI** | **t** | ***P* for t** |
| --- | --- | --- | --- | --- | --- |
| Slope | 0.0930668 | 0.2778151 | -0.5259438-0.7120774 | 0.33 | 0.745 |
| Bias | -1.631203 | 1.460562 | -4.885538-1.623132 | -1.12 | 0.290 |

Note

Abbreviation: Coef., coefficient; SE, standard error; CI, confidence interval.

We observe the *P* of the Egger’s regression asymmetry test is 0.33 > 0.05, so there isn’t significant publication bias among disease-free survivals of included trials.

**Table S2.** Sensitivity analysis for pooled disease-free survival with random-effect model

| **Study omitted** | **HR** | **95% CI (lower-bound)** | **95% CI (upper-bound)** |
| --- | --- | --- | --- |
| CALGB 49907 | 0.82288666 | 0.73604944 | 0.9199687 |
| CBCSG010 | 0.82413744 | 0.73709246 | 0.92146176 |
| CIBOMA/2004-01 | 0.8095661 | 0.71930751 | 0.91115034 |
| CREATE–X | 0.83273897 | 0.74471765 | 0.93116389 |
| FinXX | 0.82554284 | 0.73970415 | 0.92134264 |
| GAIN | 0.79670666 | 0.71159948 | 0.89199265 |
| GEICAM/2003–10 | 0.79833248 | 0.71526818 | 0.89104305 |
| SYSUCC-001 | 0.82585606 | 0.73866981 | 0.92333304 |
| TACT2 | 0.77475063 | 0.68930706 | 0.87078542 |
| USO 01062 | 0.81148175 | 0.72472612 | 0.90862273 |
| Gepar TRIO | 0.80575969 | 0.71979642 | 0.90198931 |
| EA1131 | 0.80636098 | 0.72247517 | 0.89998667 |
| Combined | 0.81134228 | 0.72855997 | 0.9035307 |

Note

Abbreviation: HR, hazard ratio, CI, confidence interval.

**Table S3.** Sensitivity analysis for pooled overall survival with random-effect model

| **Study omitted** | **HR** | **95% CI (lower-bound)** | **95% CI (upper-bound)** |
| --- | --- | --- | --- |
| CALGB 49907 | 0.74051285 | 0.63310759 | 0.86613916 |
| CBCSG010 | 0.75491916 | 0.64854017 | 0.87874731 |
| CIBOMA/2004-01 | 0.71257002 | 0.60460042 | 0.83982085 |
| CREATE–X | 0.77094835 | 0.66172071 | 0.89820576 |
| FinXX | 0.76700175 | 0.65743301 | 0.89483138 |
| GAIN | 0.74035167 | 0.63193143 | 0.86737354 |
| SYSUCC-001 | 0.74939361 | 0.64167084 | 0.87520072 |
| USO 01062 | 0.77020304 | 0.65801073 | 0.9015244 |
| EA1131 | 0.73596223 | 0.63010409 | 0.85960464 |
| Combined | 0.74945448 | 0.64687622 | 0.86829907 |

Note

Abbreviation: HR, hazard ratio, CI, confidence interval.
